# Copper Deficiency and Polyneuropathy: A Case Report

**DOI:** 10.7759/cureus.28261

**Published:** 2022-08-22

**Authors:** Hassan A Chami, Mary Ann Kirkconnell Hall

**Affiliations:** 1 Department of Medicine, Division of Hospital Medicine, Emory University School of Medicine, Atlanta, USA

**Keywords:** copper myeloneuropathy, ceruloplasmin transferrin, polyneuropathy, bariatric surgery, copper deficiency

## Abstract

Although copper plays a pivotal role in numerous physiological processes, its deficiency is virtually indistinguishable from subacute combined degeneration due to cobalamin deficiency. Moreover, the co-occurrence of deficiencies in other micronutrients and vitamins is common, making the diagnosis even more challenging. Here, we describe a case of copper deficiency in a 50-year-old woman who presented with altered mental status and bilateral upper and lower extremity weakness, numbness, and paresthesia. She was treated for cirrhosis and hepatic encephalopathy secondary to hepatic injury. While her mental symptoms improved, her physical symptoms continued to worsen, and she was transferred for further evaluation. The neurologic examination was positive for sensory neuropathy including decreased vibration/proprioception and ataxia in arms and legs; complete blood count showed pancytopenia; but infectious workup, cerebrospinal fluid analysis, autoimmune studies, and brain/spine magnetic resonance imaging were normal. A nerve conduction study showed generalized, axonal sensorimotor polyneuropathy. Micronutrient/trace element deficiency was suspected in the setting of gastric bypass surgery, and supplementation was successfully initiated. Though uncommon, clinical copper deficiency is increasingly frequently recognized in the inpatient setting, and permanent neurological damage can occur prior to diagnosis and treatment. Physicians should have an elevated clinical suspicion of copper deficiency in cases of polyneuropathy and pancytopenia in patients with a history of bariatric surgery.

## Introduction

Copper plays a pivotal role in cellular transportation, mitochondrial oxidative metabolism, neurotransmitter biosynthesis, and maintaining the structure and function of the nervous and hematopoietic systems. It is present in a wide variety of foods and is primarily absorbed in the stomach and proximal duodenum.

Copper deficiency, once termed “swayback” or “enzootic ataxia,” has been well-described in animals, particularly ruminants. Acquired copper deficiency has been recognized to cause myelopathy and neuropathy in humans relatively recently. The clinical presentation of copper deficiency is virtually indistinguishable from subacute combined degeneration due to cobalamin deficiency. Patients usually have difficulty walking, with other common symptoms including problems with balance, numbness, paresthesia (usually in the legs), and bilateral leg weakness [1]. On physical examination, patients often have reduced vibration and proprioception in the legs, spasticity, hypoesthesia in the distal portion of the hands/arms (i.e., the “stocking-glove distribution”), along with hyperreflexia, hyporeflexia, and varying plantar responses [1]. The onset of acquired copper deficiency is more common in women and those between 50 and 70 years of age [2]. Symptom progression is generally subacute (occurring over weeks to months). The literature contains reports of symptom duration prior to diagnosis of two months up to ten years [1]; this lag in diagnosis is likely attributable to providers’ unfamiliarity with copper deficiency. As noted above, copper deficiency is virtually indistinguishable from subacute combined degeneration due to cobalamin deficiency, and diagnosis can be further complicated by concurrent vitamin and micronutrient deficiencies.

Hematologic manifestations of copper deficiency such as anemia are more common than neurological symptoms [3,4]; however, myelopathy has been observed without accompanying hematologic laboratory abnormalities. Neutropenia is the cytopenia most frequently associated with copper deficiency, followed by thrombocytopenia [1]. Other rare manifestations include iron overload and/or cirrhosis, which has been described in five patients with copper deficiency myeloneuropathy [5,6]. Early diagnosis and copper supplementation generally prevent further neurologic deterioration, but improvement of neurologic signs and symptoms is variable, and most patients have some residual deficits [4,7-9].

Here, we describe a case of copper deficiency in a 50-year-old woman who initially presented with symptoms of altered mental status and bilateral upper and lower extremity weakness, numbness, and paresthesia. She was treated for cirrhosis and hepatic encephalopathy. When her mental symptoms improved, but her physical symptoms continued to worsen, she was transferred to our facility for further evaluation.

This article was previously presented as a poster at the 2021 Emory University School of Medicine, Division of Hospital Medicine Clinical Vignette Competition on September 10, 2021.

## Case presentation

A 50-year-old woman with a history of hypertension, macrocytic anemia, and morbid obesity treated with gastric bypass 19 years ago presented with bilateral upper and lower extremity weakness, numbness, and paresthesia for one month (see Table 1 for the case timeline). At baseline, she was independent and required no assistance. Three weeks prior, she had been admitted to an outside hospital with altered mental status and generalized weakness. She was diagnosed with cirrhosis and hepatic encephalopathy, for which she was treated with lactulose/rifaximin, and her mental status returned to baseline. As she began having more numbness, paresthesia, and weakness in her lower extremities, she was transferred for further evaluation.

**Table 1:** Case timeline.

Hospital day	Significant events
-19 years	Gastric bypass performed
-1 month	Onset of bilateral upper and lower extremity weakness, numbness, and paresthesia
-3 weeks	Patient admitted to an outside hospital with altered mental status and generalized weakness
0	Continued worsening of numbness, paresthesia, and weakness in her lower extremities prompts patient transfer to our facility for assessment
+4	Copper deficiency confirmed
+8	Copper supplementation started and patient discharged to a rehab facility

Clinical findings

On physical examination, she was awake and alert but was slow to respond and required frequent prompting to answer questions, and generally demonstrated poor short- and long-term memory. Neurological examination was significant for mild dysarthria and pronator drift in both arms and legs, 4/5 muscle strength in extensor and flexor muscles, absent vibratory sensation extending up to the ankles and wrists, absent proprioception in the upper and lower extremities, dysmetria on the finger to nose testing, and ataxic gait. Her deep tendon reflexes were absent over the knees and ankles and 2+ in the upper extremities. The plantar response was negative.

Diagnostic assessment

She underwent an extensive workup including cerebrospinal fluid (CSF) analysis, autoimmune studies, and magnetic resonance imaging (MRI) of the brain and spine.

She had normal thyroid-stimulating hormone, HbA1C, and tumor marker levels (alpha-fetoprotein 5.7, carcinoembryonic antigen 4.5, and cancer antigen 19-9: 28, which were ordered to assist detection of specific malignancy in the event that the patient was determined to have a paraneoplastic process). Auto-antibodies were normal except for mild elevation in extractable nuclear antigens and double-stranded DNA antibodies. A hepatitis panel was negative and she did not have signs of prior hepatitis B infection or vaccination. She was negative for HIV and had a non-reactive rapid plasma reagin test. CSF analysis and cytomegalovirus, Epstein-Barr virus, and varicella zoster virus assays were negative.

MRI of the spine did not show any evidence of spinal cord enhancement or abnormal signals. Electromyography showed severe, generalized, axonal sensorimotor polyneuropathy without radiculopathy or myopathy. Given her history of gastric bypass surgery, micronutrient/trace element deficiency was suspected. She had normal lead, zinc, mercury, vitamin B1, B6, B12, folate, and iron levels, but her serum copper and ceruloplasmin levels were slightly reduced (Table 2).

**Table 2:** Vitamin/micronutrient levels.

Vitamin/Trace element	Test result	Normal range
B1 (thiamine)	71 nmol/L	70–180 nmol/L
B6 (pyridoxine)	66 nmol/L	20–125 nmol/L
B12 (cobalamin)	780 pg/mL	180–914 pg/mL
Folate	13 ng/mL	>5.9 ng/mL
Copper	74 µg/dL	85–180 µg/dL
Ceruloplasmin	19 mg/dL	20–60 mg/dL

Given the results of the testing and her history of bariatric surgery, the patient was diagnosed with severe, generalized, axonal sensorimotor polyneuropathy secondary to copper deficiency.

Therapeutic intervention

She was started on oral replacement therapy and continued supplementation until clinic follow-up one month after discharge.

Follow-up and outcomes

The patient was discharged to follow up in the outpatient clinic. She was adherent to her medications and finished two months of replacement. She reported significant improvement but continued to experience paresthesia and mild bilateral lower extremity weakness. Despite these problems, she could independently conduct activities of daily living.

## Discussion

The daily dietary requirement for copper is relatively low, and several specific risk factors are usually associated with cases of deficiency. Hence, copper deficiency is uncommon unless these risk factors are present. In the largest case series, risk factors for copper deficiency included a history of gastric surgery (including bariatric surgery) in almost half of the cases, zinc toxicity in 16% of the cases, and malabsorption (due to conditions such as cystic fibrosis, celiac disease, and inflammatory bowel disease) in 15% of the cases [8]. No specific cause of deficiency was found in the remainder of the cases (20%). Copper deficiency after gastric surgery is presumed to result from physically decreased absorption area and inappropriate use of or nonadherence to recommended nutritional supplementation [3,4]. With an estimated prevalence of 10% among patients with a history of bariatric surgery, copper deficiency is one of the most frequently observed nutritional deficiencies among this population (after cobalamin and thiamine), but most patients have no symptoms of the deficiency [3,10,11].

The pathophysiology of myeloneuropathy from copper deficiency is not well understood. Decreased cytochrome-c oxidase activity is thought to contribute to underlying neurological dysfunction [3,9]. In laboratory findings during copper-deficient states, the total serum copper level and ceruloplasmin levels are low; 24-hour urine copper is normal or low [1].

Oral supplementation with 2 mg of copper per day is generally adequate to resolve deficiency for most patients; however, multiple treatment regimens are available, including 8 mg daily with weekly decrements over four weeks toward a maintenance dose of 2 mg daily [3,10,12,13]. Intravenous preparations have been used successfully to rapidly correct copper deficits.

While supplementation can prevent further neurologic deterioration, most patients have some residual neurological deficits. Subjective improvements in neurological symptoms have been described in as many as 50% of cases, but objective improvements are much rarer [3,4,7-9]. Delays in making the diagnosis and initiating appropriate treatment may partly explain these outcomes.

## Conclusions

Clinical copper deficiency is increasingly frequently recognized in the inpatient setting. Almost half of the cases occur following gastric/bariatric surgery. Hematologically, copper deficiency can present as anemia and neutropenia. Neurologically, it can manifest as myelopathy and peripheral neuropathy simulating subacute combined degeneration. Early diagnosis and copper supplementation generally prevent further neurologic deterioration, but improvement in neurologic signs and symptoms is variable, and most patients have some residual deficits.
